# Wellbeing and mental health amongst medical students in Canada

**DOI:** 10.1177/00207640211057724

**Published:** 2021-11-18

**Authors:** Thomas Christopher Wilkes, Thomas Lewis, Mike Paget, Johanna Holm, Nancy Brager, Andy Bulloch, Frank Macmaster, Andrew Molodynski, Dinesh Bhugra

**Affiliations:** 1 Department of Psychiatry, Cumming School of Medicine, University of Calgary, Calgary, AB, Canada; 2 Tees Esk and Wear Valleys NHS Foundation Trust, Darlington, UK; 3 Cumming School of Medicine, University of Calgary, Calgary, AB, Canada; 4 The Mathison Centre for Health Research and Education, Calgary, AB, Canada; 5 Oxford Health NHS Foundation Trust, Oxford, UK; 6 Kings College, London, UK

**Keywords:** Medical students, wellbeing, Canada

## Abstract

**Research:**

There is abundant data revealing that there is significant rate of rates of Psychiatric morbidity, psychological stress, and burnout in the medical student population. A core study group in the UK collaborated with 12 countries around the world to review medical student wellness. In this context we surveyed 101 medical students at the Cummings medical school, Calgary, Canada during the height of the COVID pandemic regarding their wellbeing and mental health.

**Results/main findings:**

Prior to medical school 27% reported a diagnosis with a mental disorder. Whilst at medical school 21% reported a mental health condition, most commonly an anxiety disorder and or depressive disorder. The most commonly reported source of stress was study at 81%, the second being relationships at 62%, money stress was a significant source of stress for 35%, and finally 10% reported accommodation or housing as stressful. Interestingly only 14% tested CAGE positive but 20% of students reported having taken a non-prescription substance to feel better or regulate their mood. Seventy-five percent of medical students met specific case criteria for exhaustion on the Oldenburg Burnout inventory 74% met criteria for the GHQ questionnaire.

**Conclusions:**

These findings confirm that medical students are facing significant stressors during their training. These stressors include, in order of frequency, study, relational, financial, and accommodation issues. Nonprescription Substance use was a common finding as well as exhaustion and psychiatric morbidity. Future interventions pursued will have to address cultural issues as well as the organizational and individual determinates of stress.

## Background

The core study group based in the United Kingdom completed phase one of an international study into medical student wellness, burnout, and substance use involving 12 countries and completed in late 2020 (Molodynski et al., 2021). The Canadian part of this group published findings in 2019 from a survey of 69 medical students’ clearly demonstrating significant morbidity and stress in this population before the Global COVID pandemic (Wilkes et al., 2019). Now there is accumulated evidence from around the globe revealing significant rates of psychiatric morbidity, psychological stress, and burnout in the medical student population (Dyrbye et al., 2006, 2008; Saipanish, 2003; Sherina et al., 2004) and that the prevalence of depression is higher than that seen in the general population (Moir et al., 2018). These issues can harm academic performance may lead to overt psychiatric conditions (Dyrbye et al., 2006), and are associated with the use of harmful substances (Ashton & Kamali, 1995; Newbury-Birch et al., 2001), stress related academic dishonesty (Rennie & Rudland, 2003), and lessened empathy (Woloschuk et al., 2004).

This current Canadian study wanted to address the aims to address the impact of the COVID pandemic on the level of psychiatric morbidity, burnout, and substance use in Calgary medical students. There is clearly an impact on education and clinical rotations when in person teaching or clinical exposure is reduced to on line exposure only. This has been described by ElHawary et al. (2021) from McGill. They published data from 13 medical schools across Canada involving 248 medical students after medical school curricula underwent major restructuring during the pandemic. Students reported a 74% reduction in the quality of their education since COVID 19 started. Additionally 58% of students found on line teaching to be inferior to in person teaching. About half of the cohort felt more depressed and lonely. Students reported less positive health behaviors after the start of the pandemic with an increase in alcohol consumption, time spent seated, and increased screen time. Students with a prior history of depression or anxiety also reported a worsening of symptoms.

Another publication by the Canadian Medical Association (CMA) prior to COVID pandemic indicated that a third of Canadian doctors were ‘burnt-out’ or depressed, and nearly 1 in 10 had thought about suicide in the past year (Vogel, 2018). According to a CMA survey of 2,547 Physicians and 400 medical residents, 30% reported high levels of burnout, 34% met criteria for depression, and 8% had thought about suicide in the last month. Thirty-seven percent of Canadian medical students meet criteria for burnout according to the Canadian Federation of Medical Students (CFMS) as published in the journal of the CMA (Glauser, 2017). This publication called for urgent action to address the current culture with regards to 70-hour clinical weeks, sleep deprivation, denial of stressors, and blind persistence with the status quo. Some schools have moved to destigmatize seeking help by having a mental health yearly ‘check-in’ with a social worker, akin to a primary-care visit. Now many Clinical services are stretched to the limit due to the COVID pandemic impact and preceptors and nurses may be even more exhausted which will impact the educational experience of medical students.

Relevant to this study on the University of Calgary medical student wellness during COVID pandemic is study by Cherak et al. (2021) from the University of Calgary. This explored the impact of the COVID 19 pandemic on 20% (540) of the 2,741 enrolled learners at the Faculty of Medicine using a cross-sectional, inter-net based survey which collected quantitative and qualitative data. This included 22% of medical students and 23% of post graduate medical residents as well as 25% of undergraduate students and 27% of graduate students. They demonstrated that learner wellness across all stages of training was negatively impacted. The importance of acknowledging equity, diversity, and inclusion, fostering psychological safety and engaging learners as active participants is key in developing wellness interventions.

## Methods

Students at the Cumming School of Medicine in Calgary, Alberta were asked to complete a one-off survey, which formed part of an international collaboration looking at mental health and wellbeing in medical students around the globe.

The aim of the survey was to quantify and characterize difficulties medical students face with regards to stressors, psychological distress, and psychiatric morbidity using standardized, reliable, and valid instruments. In the process of the survey, we aimed to encourage students to self-reflect on wellbeing and self-care. The survey included:

1. Demographic information including year of study, age, gender, educational level of parents
2. Previous mental health issues prior to medical school entry, if any
3. Short-form general health questionnaire (GHQ-12) to identify minor psychiatric disorder
4. The Oldenburg Burnout Inventory (OLBI)

Access to the survey was emailed to all current medical students at the 3-year Cumming School of Medicine in Canada. Information was provided to students regarding the nature of the study and that data collected would be anonymous. Data was stored anonymously and password protected, with only the study team having access. The students were not required to give personal identifiers as part of the survey. We used an independent, University of Calgary provisioned survey mechanism and stripped the data of any network or electronic metadata before being sent to Europe.

Pre-selected scores were used to indicate ‘caseness’ for each of the questionnaires embedded within the survey. For the CAGE questionnaire, an answer of ‘yes’ to two or more questions was used to demonstrate likelihood of problematic alcohol use. A total score above 2 was selected for the GHQ-12 in line with commonly utilized cut-off for this survey (Goldberg et al., 1997). Answers to the OLBI were scored against the dimensions of disengagement (mean >2.1) and exhaustion (mean >2.25) – thresholds shown to correspond with Maslach Burnout Inventory predictions for physician diagnosed burn-out (Peterson et al., 2008).

Additionally we planned to compare the results of the first survey to the second, we used chi-square tests to determine if key responses differed.

## Results

The results of the second survey will be presented according to the demographics, mental health findings, alcohol, and substance use and finally the GHQ and OLBI. This survey was larger than its predecessor and exceeded the sample size of 80, necessary to reach 10% of the margin of error. Any major difference with the first survey will be pointed out and discussed later.

## Demographics

There were 101 respondents, all attended Cumming School of Medicine.

About 36 (36%) reported being in Clerkship, 39 (38%) in Year 1, 26 (25%) in Year 2.

About 72 (71%) reported their gender as female, 28 (27%) as male, and 1 (1%) as other.

The most common highest education status achieved by their parents was Postgraduate degree (44%, 43%), followed by Undergraduate Degree (33%, 32%).

About 83 (82%) reported not currently working, 12 (11%) <8 hours a week, 5 (5%) 8 to 20 hours per week, and 1 (1%) more than 20 hours per week.

## Mental health

Prior to medical school, 50 (49%) had visited a professional regarding their mental health and 27 (26%) reported a diagnosis with a mental health condition. Eleven reported a previous diagnosis of a depressive disorder, 16 an anxiety disorder (including OCD), four an eating disorder, one Tourette’s, and two PTSD.

Fifteen reported a previous diagnosis of ADHD. None reported a diagnosis of Autistic Spectrum Disorder.

About 37 (36%) reported having been previously prescribed medication for their mental health. Twenty-five reported having previously been prescribed an antidepressant, 12 an ADHD medication, and four a benzodiazepine.

Whilst at medical school, 21 (20%) reported a diagnosis of a mental health condition. Nine reported a diagnosis of a depressive disorder, 12 an anxiety disorder (including OCD), 1 eating disorder, and 1 PTSD. About 37 (36%) reported they were currently seeing a professional for their mental health.

About 27 (26%) reported currently taking a medication for their mental health. Eighteen reported being prescribed an antidepressant, six an ADHD medication, two a benzodiazepine, and one a mood stabilizer.

The most commonly reported source of significant stress was study (82%, 81%), the second being relationships (63%, 62%). About 35 (34%) reported money as a significant source of stress, and 10 (9%) reported housing.

## Alcohol and substance use

About 14 (13%) tested as CAGE positive.

About 19 (18%) reported having taken a non-prescription substance to feel better or uplift their mood on at least one occasion. Six students reported weekly use and two reported daily use.

About 10 (9%) reported having used a medication to enhance concentration, study, or academic performance. Eight reported using ADHD medications. Two people used these medications daily.

About 40 (39%) reported having previously used cannabis, eight (8%) ecstasy, five (5%) amphetamines, one (1%) ketamine, three (3%) cocaine, two (2%) opiates, and nine (9%) another substance.

Three students (3%) reported someone else had been worried about their substance use, while five (5%) reported concern themselves about substance use.

## GHQ and OLBI

About 75 (74%) met specified case criteria for the GHQ questionnaire.

For the OLBI questionnaire, 62 (61%) met specified case criteria for disengagement, and 76 (75%) met specified case criteria for exhaustion.

**Table:** 

Year of study	Average of GHQ	Average of OLBI
1	2.335586	2.484797
2	2.282051	2.480769
3	2.293011	2.4375

## Discussion

At the Cumming School of Medicine in the University of Calgary, Canada, we have approximately 470 medical students. This necessitates a sample of 80 to meet a 95% CI with a 10% margin of error. We are pleased that this sample size of 101 is bigger and the findings are therefore more robust. This represents a 21% response rate and we recognize a higher number of responses from female students in their clerkship year, which may reflect a response bias. Nevertheless, there are some general findings which are worthy of note.

The findings of the pre COVID survey and COVID survey on medical student wellness, psychiatric morbidity, and burnout are similar (see Table 1). The level of exhaustion in our medical students is significant (74%) and similar to the pre-COVID survey. A majority (81%) of the surveyed group recognized academic pressures as a source of stress, and relationship (62%) and money worries (35%) and housing (10%) were also notable which may reflect the challenges inherent in residency applications. Financially, our students at University of Calgary have higher than average debt and this was a significant stressor for students in the last phase of the program in the first survey (60% of respondents in clerkship vs. 26% in pre-clerkship).

**Table 1.** Summary of χ^2^ tests (1, *N* = 170) comparing the two survey time points.

Question	χ^2^	*p*-Value
Visited a professional regarding their mental health	0.1607	.69
Diagnosis with a mental health condition	0.0088	.93
Previously prescribed medication for their mental health	1.0759	.30
Diagnosis of a mental health condition	0.6501	.42
Taking a medication for their mental health	0.0134	.91
Significant stress – study	0.5405	.46
Significant stress – relationships	0.0340	.85
Significant stress – money	2.3594	.12
CAGE positive	1.7981	.18
Taking a non-prescription substance to feel better or uplift their mood	0.0555	.81
Used a medication to enhance concentration, study, or academic performance	0.0027	.96
Cannabis use	0.1974	.66
Meeting specified case criteria for the GHQ questionnaire	0.0265	.87
OLBI – disengagement	0.0991	.75
OLBI – exhaustion	0.6706	.41

*Note.* With correction for multiple comparisons, *p* < .003 to be considered significant. Bonferoni test was used with the multiple comparisons.

Secondly, the high prevalence of anxiety and depression in our survey speaks to the issue of the importance of timely access to mental health counseling and support from the student wellness office and the larger community. Our results also suggest a need for advice for financial planning. This finding of increasing numbers university students in general, not just medical students struggling with anxiety and depression has been well documented by Haidt (2018) and Twenge (2017) Both our surveys reported a pre-university prevalence of around 26% for diagnosed mental health disorder (28% in the first survey and 27% in the second). Our findings in this report were consistent with our previous survey. Similar numbers reported currently taking medication for their mental health. Taken together these findings suggest there is a significant need for timely access to medical services for prescribing medications to avoid self -medicating.

Nevertheless, it is worrisome to see high CAGE positive rates at 13.9%, however this is lower than the first survey results of 22%. This maybe because during the COVID pandemic lock down there was less chance to socialize or participate in sports events. Nevertheless, this highlights the importance of informed counseling about the risks and benefits of alcohol use as well as for a variety of other substances. The latter now includes legalized cannabis which is widely available, but also the more serious, illegal drugs such as ecstasy, psilocybin, amphetamines, ketamine, cocaine, and opiates.

When compared to the international study of Molodynski et al. (2021) from 12 countries the Calgary survey of medical students shows similar rates of psychiatric morbidity ranking about sixth out of 12. But exhaustion and burnout measurements were to be found in the lowest quartile the 12 countries. Hong Kong demonstrated some of the highest rates on all the metrics which may suggest intense family and cultural pressures to succeed. Finally, Cannabis use was legalized in Canada in 2018 and its use in medical students was higher in our survey, in the top quartile, below that of Portugal and Brazil. The CAGE questionnaire found Canada was about seventh out of the 12. Suggesting that Cannabis in Canada has become the preferred substance of use.

In the medical culture there is a pressing need to address burnout – the emotional exhaustion, depersonalization and the resulting reduced sense of personal accomplishment – this is well described by Maslach et al. (2001) and others. The question now arises how best to address these issues – both individually and from an organizational perspective. The purely individual approach can involve counseling, such as third generation cognitive behavioral (CBT) interventions including Mindfulness based CBT. These approaches however will be inadequate unless organizational and cultural issues are also addressed.

These organizational and cultural factors may be further complicated by a generational effect; there is evidence by Jean Twenge suggesting a cohort effect for the i GEN (those students born in and after 1995) demonstrating higher rates of Anxiety, depression, and suicide. Lukianoff and Haidt (2018) discuss how six variables come together; increasing prevalence rates of anxiety and depression due to the digital age, increasing polarization of society, decreased free play time, paranoid parenting, a huge safety culture impacting the size of university bureaucracy, and greater interest in in equity, inclusion, and social justice. They suggest that these have promoted a ‘victimhood’ culture in universities with its attendant emotional reasoning, micro-aggressions and there has been significant concept creep with the term violence being applied to contradictory views to the orthodox political views of social justice. Now freedom of speech is challenged on the grounds it can make people uncomfortable and the telos of some universities is social justice rather than the pursuit of truth through reflective enquiry. They emphasized that students should be taught cognitive behavioral therapy to promote critical thinking and to cultivate a stoic attitude as illustrated by skills of a good cognitive therapist but also seen in history several millennia ago by Marcus Aurelius, ‘Our life is what our thoughts make of it’. Epictetus, ‘It is not events themselves that trouble us, but what we make of them’. Shakespeare in Hamlet, ‘there is nothing good or bad but thinking makes it so’ and finally the Buddha sums it up with ‘Our life is the creation of our mind’. Further qualitative exploration is clearly needed to understand the socio-cultural and political pressures medical students face at University.

In Alberta there have been active partnerships with the Alberta Medical Association (AMA) and the University Calgary Cummings Medical School to look at burnout through both educational activities and social activities including lunch and learn activities addressing issues of racism in medicine and the decolonization of the medical curriculum. The AMA also promotes access to timely mental health services through the ‘Physician Wellness Program’ and the Student Wellness Office. Hopefully this destigmatizes mental illness and the need to seek help or explore underlying factors that may undermine resilience and competence. Perhaps at this point it is a timely reminder that other disciplines, such as social work, have published work on the role of the family of origin in vocational choice. Lackie (1983) illustrated how the personal psychology of the typical healer included the parentified child, the over responsible member of the family, the mediator or the go between or the burden bearer. Of course, the child cannot succeed in these roles so they then carry chronic anxiety and guilt and feel compelled to respond repeatedly regardless of whether this is the most appropriate response. Similarly Hanna (2009) emphasized the relationship between the False Self compliances and the motivation to become a professional helper tell us that the false self, as described by Donald Winnicott’s, merges with the ideas of vocation, in particular the helper as during their early childhood experiences they perceive that they are needed to maintain the parental narcissistic equilibrium.

There has been a promotion of a student ombudsman for the purpose of voicing concerns and providing advocacy regarding the environmental and cultural issues of tolerance, diversity, and professionalism. There is already robust evidence that CBT and mindfulness-based meditations help manage depression and anxiety. When applied to the workplace for all students and staff, involvement with activities such as yoga sessions at retreats or use of technology for film sessions and relaxation all can result in a sense of meaning, gratitude, and fulfilment. However the COVID pandemic has limited many of the social supports and connections that have been used in the past. In this context the University of Calgary has been involving medical students in STRIVE sessions, (Simulated Training for Resilience in Various Environments), developed in conjunction with the Canadian Federation of medical students and the Canadian Armed Forces military. STRIVE was developed as road to mental readiness program training and reinforced with experiential learning through practical simulation training. This focused on the big four such as smart goal setting, visualization, positive self-talk, and box breathing with progressive muscle relaxation as outlined by Smith et al. (2020). However, the larger organizational interventions such as addressing equity and diversity are often seen as more important due to the risk of isolation and fragmentation of students in educational sections. This can be addressed by having retreats that share an inter-locking mission statement and that work within identified organizational structures, thus addressing the antecedents of burnout and stress through the promotion of a positive culture of personal agency and resilience.

## Conclusions

This survey of wellbeing and mental health of medical students at Cumming School of medicine at the University of Calgary, Canada during the COVID pandemic clearly demonstrates the need for support to mitigate significant stress (74%) and mental ill health (27%) that students experience during their training. Based upon the prevalence of the sources of stress for medical students, study (81%), relationships (62%), Financial (34%), Housing (9%), and CAGE positive rates of 13%. Particular attention should be focused upon stress management techniques such as mindfulness based CBT rather than just social and sports activities which can encourage inappropriate use of substances. These findings speak to the need for timely access to mental health services with evidence based treatments and the importance of an awareness of the organizational and cultural attitudes such as the denial of stress and personal needs during an arduous medical education and thus promote a healthier work life balance. Further research and cultural exploration is needed to understand the diverse pressures medical students face.
